# Increased human immunodeficiency virus viral load with cerebral infarction due to varicella zoster virus vasculopathy on treatment with bictegravir/emtricitabine/tenofovir alafenamide suspension: a case report and literature review

**DOI:** 10.1186/s12981-023-00547-7

**Published:** 2023-07-30

**Authors:** Kazuhiro Ishikawa, Fujimi Kawai, Nobuyoshi Mori

**Affiliations:** 1 Department of Infectious Diseases, St. Luke’s International Hospital, 9-1, Akashi-cho, Chuo-ku, Tokyo, 104-0044 Japan; 2 Library, Center for Academic Resources, St. Luke’s International University, 10-1, Akashi-cho, Chuo-ku, Tokyo, 104-0044 Japan

**Keywords:** Bictegravir/tenofovir alafenamide/emtricitabine suspension, Doltegravir/abacavir/lamibudine suspension, Human immunodeficiency virus, Acquired immunodeficiency disease syndrome, Varicella-zoster virus, Vasculopathy

## Abstract

**Background:**

Varicella-Zoster virus (VZV) vasculopathy occasionally occurs in immunocompromised patients and is difficult to treat. The risk factor and optimal therapy remain unclear. Patients with human immunodeficiency virus (HIV) and dysphagia or difficulty in oral intake receive antiretroviral therapy (ART) suspension. However, there remains little evidence regarding ART suspension.

**Case presentation:**

We experienced a case of a 55-year-old man diagnosed with HIV and severe multiple cerebral infarctions due to VZV vasculopathy. We started on bictegravir/tenofovir alafenamide/emtricitabine (BIC/TAF/FTC) and acyclovir (ACV), and prednisone. He was started on BIC/TAF/FTC suspension because of deteriorated swallowing. The HIV viral load was increased; however, no drug-resistance genes were detected. We successfully treated him with doltegravir/abacavir/lamibudine suspension. We performed two literature reviews of the administration of BIC/TAF/3TC suspension and VZV vasculopathy in patients with HIV. Three cases of BIC/TAF/3TC suspension were considered treatment failures. Recent history of VZV infection and a CD4 count under 200 μL may be risk factors for VZV vasculopathy. The effective treatment may be using steroid and ACV; however, treatment duration could differ.

**Conclusions:**

BIC/TAF/FTC suspension administration may be unstable, and treating ACV and steroid may be optimal therapy for VZV vasculopathy; however, the evidence level is low.

**Supplementary Information:**

The online version contains supplementary material available at 10.1186/s12981-023-00547-7.

## Background

Bictegravir (BIC) can be administered as a single-tablet regimen and in combination with emtricitabine (FTC) and tenofovir alafenamide (TAF). BIC/FTC/TAF is among the most frequently prescribed antiretroviral therapy (ART), given its simplicity, good safety profile, and high genetic barrier to resistance [1]. However, there are safety concerns regarding BIC/FTC/TAF therapy among patients unable to swallow an intact tablet, given the limited data on suspension formulations. BIC/FTC/TAF manufacturer’s labeling does not indicate whether the tablet could be converted to suspension [2].

Varicella-zoster virus (VZV) causes chickenpox. The virus then becomes latent in ganglionic neurons. With higher age or an immunocompromised host, the VZV reactivates to cause herpes zoster (shingles). VZV causes severe neurological complications—such as vasculopathy, meningoencephalitis, spinal encephalitis, myelopathy, and postherpetic neuralgia—in immunocompromised hosts, such as patients with Human Immunodeficiency Virus (HIV) [3]. In the case series of 30 patients [4], clinical characteristics and management for almost non-HIV-infected patients are shown; however, the risks factor and optimal therapy for patients with HIV remain unclear.

We present a patient with HIV and severe VZV vasculopathy who presented with increased HIV viral load and worsened clinical course after BIC/FTC/TAF suspension administration. We performed two literature reviews of the administration of BIC/TAF/3TC suspension and VZV vasculopathy in patients with HIV to collect more evidence.

## Case presentation

**Fig. 1:**
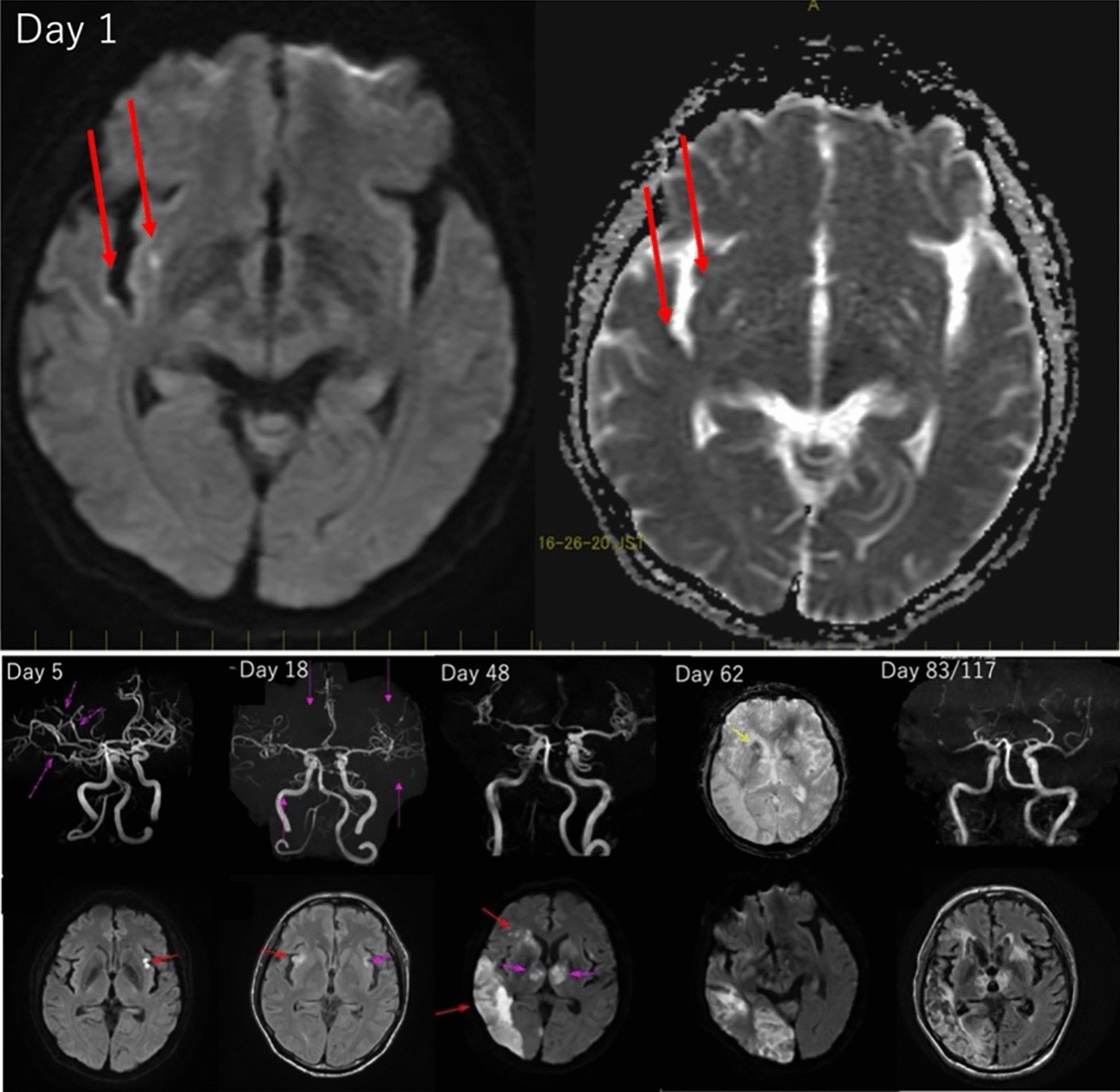
The comparative findings of the Brain MRI. The DWI revealed a high intensity in the right subinsular cortex and right temporal lobe cortex (left, on Day 1). The ADC map revealed a signal loss in the same area (right, on Day 1). The MRA scans are shown in the top row from Day 5 to Day 117; however, T2* images were obtained from day 62. The DWIs are shown in the bottom row. The scans revealed multiple cerebral infarctions and blood vessel narrowing over time. On day 62, hemorrhagic infarction was observed at T2*. On day 117, there were no changes, with a similar infarction area being observed as that on day 83. *MRI* magnetic resonance imaging, *DWI* diffusion-weighted imaging, *MRA* Magnetic resonance angiography

**Fig. 2:**
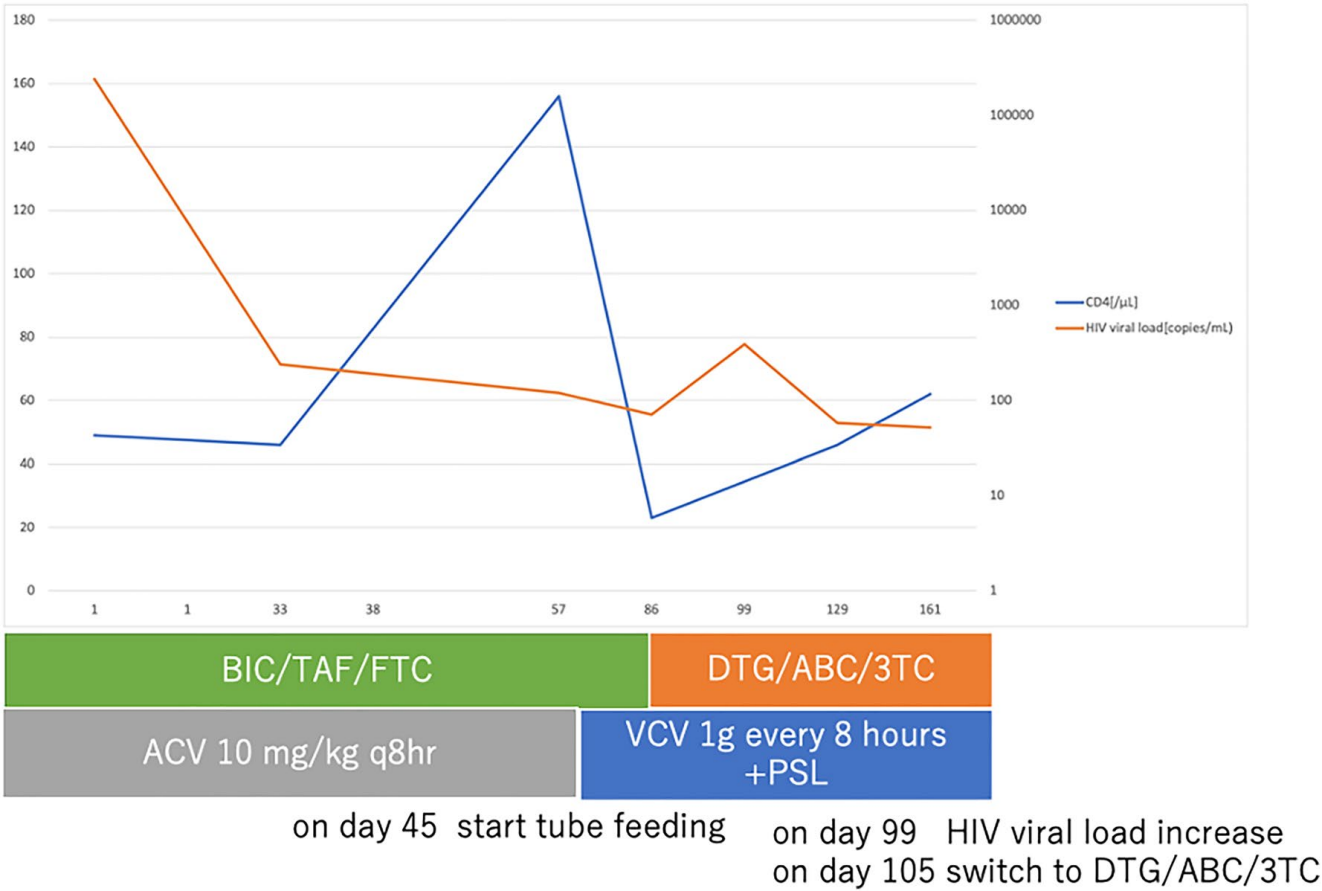
Clinical course of our case. BIC/TAF/FTC, bictegravir/emtricitabine/tenofovir alafenamide; DTG/ABC/3TC, dolutegravir/abacavir/lamivudine; *ACV* acyclovir, *VCV* valacyclovir, *PSL* prednisone

A 55-year-old homosexual man with a history of type 2 diabetes mellitus presented with herpes zoster in the left anterior thoracic area 3 months before admission. Two weeks before admission, he was diagnosed with HIV and started on BIC/TAF/FTC. On admission day, he visited our hospital complaining of high fever, headache, and consciousness disorder. His vital signs were as follows: level of consciousness, E4V4M6 in the Glasgow Coma Scale; body temperature, 36.4 °C; blood pressure, 147/97 mmHg; heart rate, 109 min; respiratory rate, 26 min; oxygen saturation at room air, 99%. Physical examination revealed no neck rigidity, no oral lesion, clear bilateral auscultation, and no skin rash or raised patches. The laboratory test results were as follows: white blood cell count, 5200 μL (lymphocytes, 810 μL; CD4, 49 μL; CD4/8, 0.12); blood glucose level, 153 mg/dL; HIV viral load, 2.4 × 10^5^/mL; cryptococcal antigen, negative; and no ART-resistant HIV mutation. The cerebrospinal fluid (CSF) test findings were as follows: initial pressure, 15 cmH_2_0; cell count, 124 μL (mononuclear cells: 88.7%); protein levels, 388 mg/dL; glucose levels, 87 mg/dL; VZV polymerase chain reaction, 9.4 × 10^4^ copies/mL; and cryptococcal antigen, negative. The CSF culture was negative. Contrast-enhanced magnetic resonance imaging (MRI) of the brain revealed cerebral infarctions in the subcortical areas (Fig. 1). He was diagnosed with cerebral infarction caused by VZV vasculopathy and was started on acyclovir (ACV) 10 mg/kg at 8 h intervals. On day 18 of hospitalization, MRI revealed worsening of the cerebral infarction (Fig. 1). There was a subsequent gradual deterioration of his swallowing function due to worsening neurological symptoms. A CSF lumbar puncture did not reveal a VZV load on day 38. He was started on tube feeding on day 45. Further, there was no BIC/TAF/FTC administration or gastrointestinal symptoms, including diarrhea. The HIV viral load decreased to 86 copies/mL on day 86 but increased to 390 copies/mL on day 99. No drug-resistance genes were detected. He was started on prednisone from day 57 (first 3 days 1 g/day, and thereafter 1 mg/kg). MRI revealed a temporal progression of the multiple cerebral infarctions (on days 48 and 62; Fig. 1). The patient was switched to Doltegravir/Abacavir/Lamivudine (DTG/ABC/3TC) on day 105. The findings of the MRI on day 117 indicated multiple cerebral infarctions progression cessation compared to the findings on day 83 (Fig. 1). On day 161, the HIV viral load was 52 copies/mL with 61 μL of CD4, and he was transferred to another hospital. The clinical course of our case is shown in Fig. 2.

### Literature review

We reviewed BIC/TAF/FTC suspension and VZV vasculopathy in patients with HIV using database records (PubMed, Embase, and Ichushi until Oct 20, 2022) and extracted information from these articles (Additional file 1: Figs. S1, S2). Search terms are enlisted in the Additional file, Additional file.doc. Finally, we extracted 9 cases of BIC/TAF/FTC suspension and 77 cases of VZV vasculopathy in patients with HIV, including our case. Additional file 1: Tables S1, S2 show the clinical characteristics of the published cases.

There were three cases of increased viral load and two cases (one case where BIC/TAF/FTC was the initial) of developed viral mutations. In the literature on VZV vasculopathy in patients with HIV, the mortality was higher before 1999 (29 cases) than after 2000 (48 cases) (79% vs. 21%). This may be because, before ART introduction, VZV diagnosis was mostly based on autopsy. Our analysis of the literature since the year 2000 shows that the median age (range): 35.5 (5–62) years; male: 65%; CD4(/μL) (median 56.5), distribution: > 200, 15%; 100 < CD4 ≤ 200, 20%; ≤ 100, 65%: a recent history of VZV; no VZV infection or more than 1 year, 46%, on admission, 21%; within 3 months, 18%; between 3 and 6 months, 8%; between 6 months and 1 year, 8%: treatment in neurological symptom improvement or stable; ACV + steroid, 82%; ACV alone, 65%: duration of treatment, excluding death; within 2 weeks, 4 cases; 3–4 weeks; 5 cases; > 1 month, 9 cases: outcome; improved neurological findings, 27 cases; residual neurological deficit, 2 cases: death, 10 cases) (Table 1).

**Table 1:** Results of a literature review of 48 patients with HIV with VZV vasculopathy complicated by cerebral infarction since 2000

Characteristic	n = 48
Median age (range)	35.5 (5–62)
Male, n(%)	31 (65%)
Median CD4 count (/μL) (range)	56.5 (1–700)
> 200, n(%)	6 (15%)
$100<\mathrm{CD}4\le$ 200, n(%)	8 (20%)
$\le 1$ 00, n(%)	26 (65%)
Not reported, n	8
The date of the previous VZV infection	
VZV infection between 6 months and 1 year, n (%)	3 (8%)
On admission, n (%)	8 (21%)
Within 3 months, n (%)	7 (18%)
Between 3 and 6 months, n (%)	3 (8%)
No VZV infection or more than 1 year, n (%)	18 (46%)
Not reported, n	9
Management	
ACV + steroid clinical improvement or stable (%)	82%
ACV clinical improvement or stable (%)	65%
Duration of the treatment without death within 2 weeks, n	4
3–4 weeks, n	5
More than 1 month, n	9
Not report treatment duration, n	12
Prognosis-clinical improvement/deterioration/death/not reported, n	27/2/10/4

*VZV* Varicella-zoster virus, *HIV* human immunodeficiency virus, *ACV* acyclovir

## Discussion

This article presents a patient with HIV who had severe multiple cerebral infarction due to VZV vasculopathy and failed treatment with BIC/TAF/FTC suspension. Although the manufacturer’s labeling indicates the safety of BIC/TAF/FTC suspension [2], there are three reported cases of failed treatment, including our case [5, 6]. Two of these cases, including ours, did not use other ART regimens [6]. Unlike our case, the two cases showed viral mutations [5, 6] We switched to DTG/ABC/3TC because the randomized controlled trials have shown that DTG/ABC/3TC has no pharmacokinetic problems when administered in suspension form to healthy adults [7]. Although Tenofovir disoproxil fumarate suspension is not listed by the FDA, the safety serum concentration level has been reported [8]. However, the SOLUBIC study reported a decrease in plasma TAF levels (Area Under the Curve: AUC 86%, 90% confidence interval (CI) [82–91], C_max_ 70%, 95% CI [63–78]) [9]. In this study, bioequivalence was met if the 90% CIs of the AUC and Cmax were within 80–125% of the reference. We could not perform therapeutic drug monitoring due to limited facilities; however, the effectiveness of BIC/FTC/TAF suspension remains unclear; and safer ART suspensions are required to avoid difficulties in swallowing or tube administration.

**Fig. 3:**
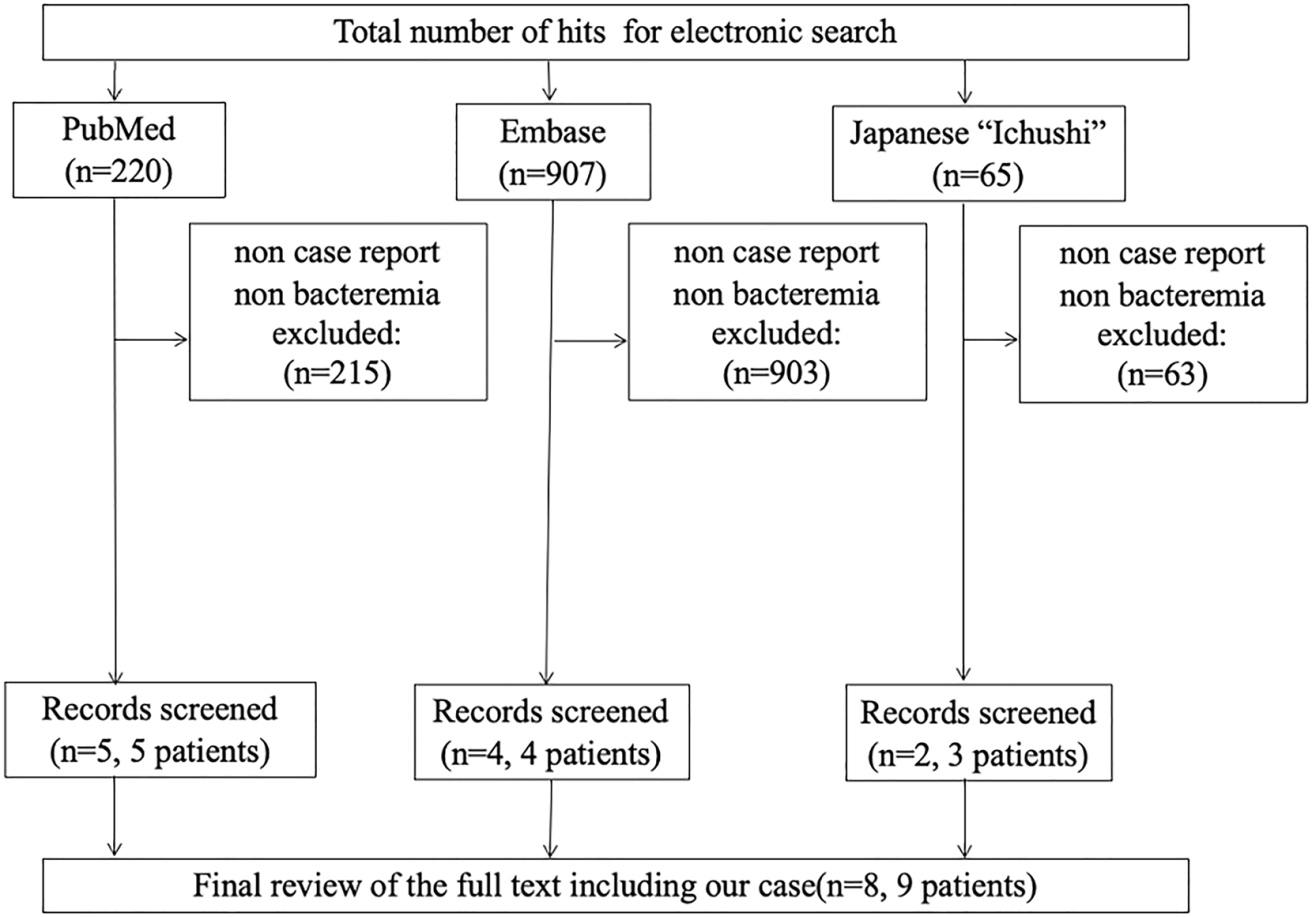
The flow chart of BIC/TAF/FTC suspension depicts the systematic review process of this study

**Fig. 4:**
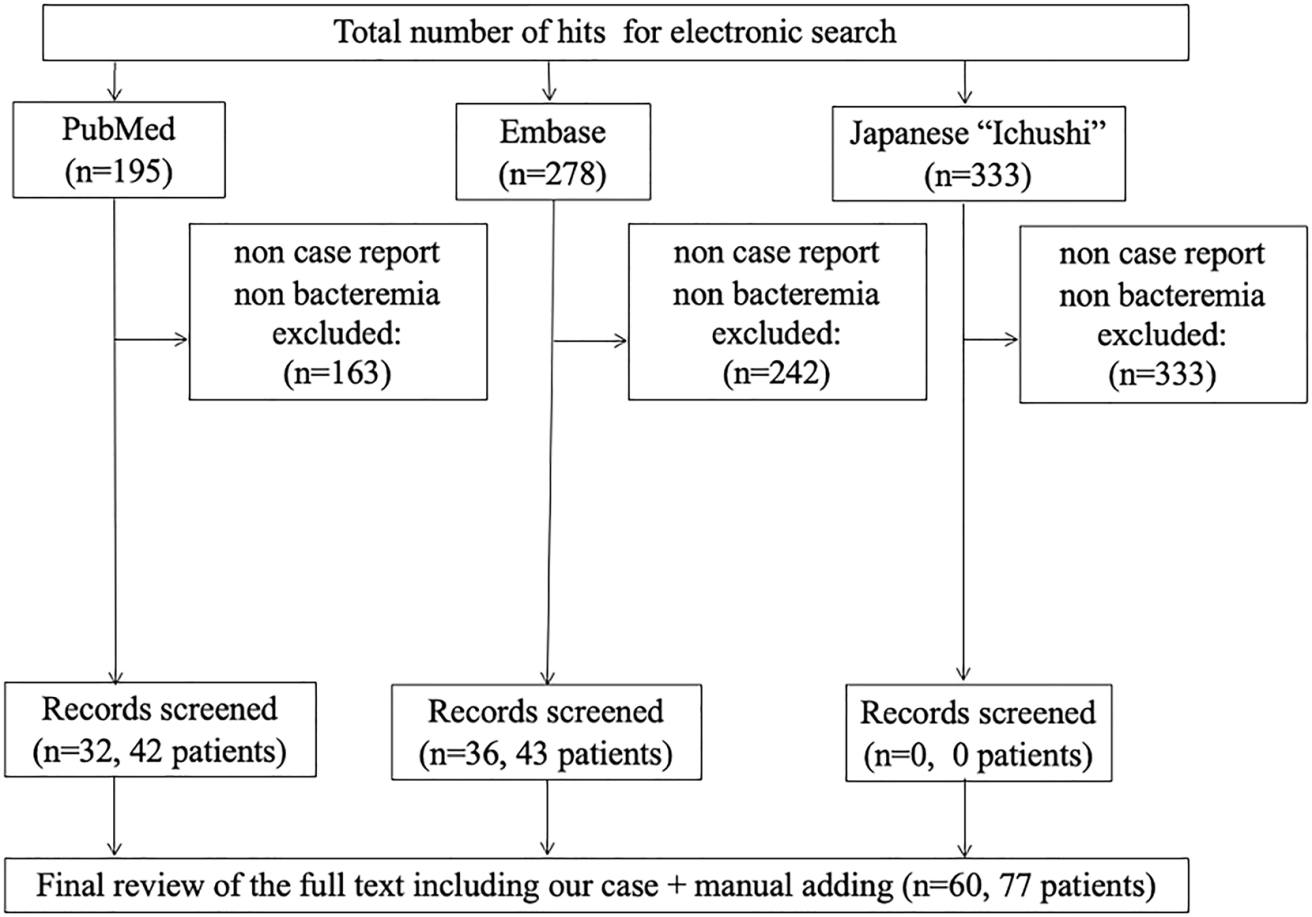
Flow chart of VZV vasculopathy in patients with HIV depicts the systematic review process of this study

In general, patients with herpes zoster are known to have significantly higher cerebral infarction within 1 year [10]. From our review, we propose that we need a careful follow-up of the neurological symptoms in patients with HIV who started ART after a recent history of VZV infection at under 200 μL in CD4 counts. Experts have recommended administering 14 days course of ACV with oral prednisone. The case series recommended a more effective treatment regimen of ACV + steroid than ACV [4]. In our review, ACV + steroid for neurologic deficits in patients with HIV were more improved or stabilized than ACV alone. We could not find the optimal duration of treatment in our review. This needs to be clarified in a further prospective study (Figs. 3, 4).

## Conclusions

Patients with HIV with CD4 counts under 200 μL during or after treatment for herpes zoster should be followed carefully for neurological symptoms. DTG/ABC/3TC may be considered over BIC/TAF/FTC in the case of suspension administration.

## Supplementary Information

**Additional file 1: Table S1** Table showing the process of the literature review regarding crushed BIC/TAF/FTC. **Table S2** Table showing the process of the literature review regarding VZV vasculopathy with HIV patient. **Figure S1.** The process of the literature review regarding crushed BIC/TAF/FTC. **Figure S2.** The process of the literature review for Varicella-zoster virus vasculopathy with stroke in patients with HIV

## Data Availability

The data that support the findings of this study are openly available.

## Abbreviations

HIV: Human immunodeficiency virus
ART: Antiretroviral therapy
VZV: Varicella-zoster virus
MRI: Magnetic resonance imaging
CSF: Cerebrospinal fluid
BIC: Bictegravir
FTC: Emtricitabine
TAF: Tenofovir alafenamide
CI: Confidence interval
ACV: Acyclovir
AUC: Area under the curve

## References

[1] Deeks ED (2018). Bictegravir/emtricitabine/tenofovir alafenamide: a review in HIV-1 infection. Drugs.

[2] Biktarvy® (BIC/FTC/TAF) Crushing or splitting of tablets. https://www.askgileadmedical.com/docs/biktarvy/biktarvy-crushing-or-splitting-of-tablets

[3] Gilden D, Nagel MA, Cohrs RJ, Mahalingam R (2013). The variegate neurological manifestations of varicella zoster virus infection. Curr Neurol Neurosci Rep.

[4] Nagel MA, Cohrs RJ, Mahalingam R, Wellish MC, Forghani B, Schiller A (2008). The varicella zoster virus vasculopathies: clinical, CSF, imaging, and virologic features. Neurology.

[5] Lozano AB, Chueca N, de Salazar A, Fernández-Fuertes E, Collado A, Fernández JM (2020). Failure to bictegravir and development of resistance mutations in an antiretroviral-experienced patient. Antiviral Res.

[6] Rowe SM, Clary JC, Drummond M, Derrick C, Sanasi K, Bookstaver PB (2022). Increased viral load in a hospitalized patient on treatment with crushed bictegravir/emtricitabine/tenofovir alafenamide: a case report and review of the literature. Am J Health Syst Pharm.

[7] Roskam-Kwint M, Bollen P, Colbers A, Duisenberg-van Essenberg M, Harbers V, Burger D (2018). Crushing of dolutegravir fixed-dose combination tablets increases dolutegravir exposure. J Antimicrob Chemother.

[8] Lalley-Chareczko L, Clark D, Zuppa AF, Moorthy G, Conyngham C, Mounzer K (2017). A case study of chewed Truvada((R)) for PrEP maintaining protective drug levels as measured by a novel urine tenofovir assay. Antivir Ther.

[9] Hocqueloux L, Lefeuvre S, Bois J, Brucato S, Alix A, Valentin C (2022). Bioavailability of dissolved and crushed single tablets of bictegravir, emtricitabine, tenofovir alafenamide in healthy adults: the SOLUBIC randomized crossover study. J Antimicrob Chemother.

[10] Kang JH, Ho JD, Chen YH, Lin HC (2009). Increased risk of stroke after a herpes zoster attack: a population-based follow-up study. Stroke.

