# Supplementary material for: Increased human immunodeficiency virus viral load with cerebral infarction due to varicella zoster virus vasculopathy on treatment with bictegravir/emtricitabine/tenofovir alafenamide suspension: a case report and literature review
Source: AIDS Res Ther. 2023 Jul 30;20:53. doi: 10.1186/s12981-023-00547-7 (PMC10388448; doi:10.1186/s12981-023-00547-7)
Supplement: Supplementary file 1 — Additional file 1: Table S1 Table showing the process of the literature review regarding crushed BIC/TAF/FTC. Table S2 Table showing the process of the literature review regarding VZV vasculopathy with HIV patient. Figure S1. The process of the literature review regarding crushed BIC/TAF/FTC. Figure S2. The process of the literature review for Varicella-zoster virus vasculopathy with stroke in patients with HIV [file 12981_2023_547_MOESM1_ESM.docx]

**Appendix A. Keywords to search the case report of administering crushed BIC/TAF/FTC**

A literature search was conducted

- PubMed (October 20, 2022)

bictegravir

- Ichushi (October 20, 2022)

(Bictegravir/TH or Bictegravir [Japanese]/AL) or (("Bictegravir-Emtricitabine-Tenofovir Alafenamide"/TH or Bictarvy[Japanese]/AL))

- Embase (October 20, 2022)

'bictegravir'/exp OR bictegravir

**Appendix B. Keywords to search the case report of Varicella-zoster virus vasculopathy with stroke in HIV patient**

A literature search was conducted

- PubMed (October 20, 2022)

( (varicella zoster virus or ((varicella zoster virus)/TW)) or( VZV or VZV/TW) ) and ((vasculopathy or vasculopathy/TW) or (stroke or stroke/TW) or (cerebral or cerebral/TW)) AND ( (HIV or HIV/TW) OR (AIDS or AIDS/TW) or (human immunodeficiency virus) or (acquired immunodeficiency syndrome))

- Ichushi (October 20, 2022)

#1 "vasculopathy/AL or (central nerve system[Japanese]/TH or or/AL and cerebral/AL or CNS/AL) or (stroke[Japanese]/TH or or/AL and cerebral infarction[Japanese]/TH or stroke/AL) or (vasculitis[Japanese]/TH or vasculitis/AL)"

#2 ((chicken pox[Japanese]/TH or varicella/AL) or (shingles[Japanese]/TH or zoster/AL))

#1 and #2

- Embase (October 20, 2022)

#1. vzv OR 'varicella zoster virus' OR 'varicella'

#2. 'vasculopathy' OR 'vasculitis' OR 'stroke' OR 'cerebral'

#3. 'hiv' OR 'human immunodeficiency virus' OR 'aids' OR 'acquired immunodeficiency syndrome'

 #4. #1 AND #2 AND #3

Table S1 Table showing the process of the literature review regarding crushed BIC/TAF/FTC

| No | author | age  (years), Sex | underlying diseases | exposer to other ARV before BIC/TAF/FTC | BIC/FTC/TAF administration notes | baseline HIV viral load | baseline CD4 cell count | follow up HIV viral load | follow up  CD 4 cell count | mutations developed | outcome |
| --- | --- | --- | --- | --- | --- | --- | --- | --- | --- | --- | --- |
| 1 | Fulco, 2020 [1] | 64,  M | esophageal cancer | DTG/ABC/3TC | crushed and dissolved BIC/ FTC/TAF using PEG | 501 copies /mL | 440 cells/μL | undetectable at 4 and 10 months via PEG | 371 cells/μL at 4 months via PEG | NA | viral load undetectable |
| 2 | Lozano, 2020[2] | 39,  F | PML | LPV/r, TDF, FTC,ABC, RAL | crushed and dissolved BIC/ FTC/TAF using NG tube | 1,084 copies/ mL | 134 cells/μL | 10,232 copies/ mL at 6 weeks via NG tube | NA | M184V, L74I, R263K | viral load increasing and mutation developed |
| 3 | Ferrández, 2021[3] | 52,  F | none | EVG/cobi/FTC/TAF | BIC/FTC/TAF tablet in orange juice before swallowing | undetectable | 370 cells/μL | undetectable  at 10 months | 370 cells/μL at 10 months | NA | viral load undetectable |
| 4 | Sarah M Rowe, 2022[4] | 43,  M | none | none | crushed and dissolved BIC/ FTC/TAF using PEG tube | 5,887 copies/mL | NA | 8,047 copies/mL  at 5 weeks | NA | E157Q, V118I | viral load increasing and mutation developed |
| 5 | Roa PE, 2021[5] | 78,  M | colon cancer with laparoscopic sigmoid colectomy,  pancreatic cancer | EFV, FTC and tenofovir disoproxil , RPV/FTC/TDF | crushed BIC/FTC/TAF using PEG | undetectable | NA | undetectable  at 7 months | NA | NA | viral load undetectable |
| 6 | Rie M, 2021[6] | 60's,  M | PML, HIV encephalopathy | none | BIC/FTC/TAF via PEG | undetectable | NA | NA | NA | NA | BIC trough 0.96μg/mL, 0.91μg/mL more than EC95(0.17μg/mL) |
| 7 | Rie M, 2021[6] | 70's,  M | DCM, septic shock, CHF | DTG+DOR | BIC/FTC/TAF via NG tube | 459 copies/mL | NA | NA | NA | NA | BIC trough 1.18μg/mL, 2.99μg/mL more than EC95(0.17μg/mL) |
| 8 | K. morishita, 2020 [7] | 60's,  F | none | DRV＋RTV＋ABC/3TC | crushed BIC/FTC/TAF | 270, 000 copies/mL | 31/μL | 46 copies/mL | 231 cells/μL | NA | viral load undetectable, BIC concentration 1.148μg/mL |
| 9 | our case, 2020 | 55,  M | diabetes, VZV vasculopathy | none | crushed and dissolved BIC/ FTC/TAF using PEG | 240 copies/mL | 46 cells/μL | 156 copies/mL 24 days after PEG administration | 120 cells/μL 24 days after PEG administration | none | viral load increasing and mutation developed |

Abbreviation: BIC/TAF/FTC, bictegravir/emtricitabine/tenofovir alafenamide; M, male; F, female; PML, progressive multifocal leukoencephalopathy;

HIV, human deficiency virus; HCV, hepatic C virus; IVD, intravenous drug user; CHF, congestive heart failure; DCM, dilated cardio myopathy; VZV, varicella-zoster virus; DTG/ABC/3TC, dolutegravir/abacavir/lamivudine; LPV/r, Lopinavir/Ritonavir; TDF, tenofovir disoproxil fumarate; FTC, emtricitabine; ABC, abacavir; RAL, raltegravir; EVG/cobi/FTC/TAF, elvitegravir/cobicistat/emtricitabine/tenofovir alafenamide;

EFC, efavirenz; RPV/FTC/TDF, rilpivirine/tenofovir disoproxil fumarate/emtricitabine; 3TC, lamivudine; DTG, dolutegravir; DOR, doravirine;

DRV, Darunavir; RTV, Ritonavir; ABC/3TC, abacavir/lamivudine; PEG, percutaneous endoscopic gastrostomy; NG, nasogastric; NA, not available; EC, effective concentration

Table S2 Table showing the process of the literature review regarding VZV vasculopathy with HIV patient

| No | author | age | underlying  disease | previous or current  VZV history | CD4(/μL) /  HIV viral load (copies/ml) | duration of time since ART started | diagnosis of  VZV | complications of  other than VZV vasculopathy | duration of treatment | ACV  (including FOS, GCV GACV) | steroid | prognosis |
| --- | --- | --- | --- | --- | --- | --- | --- | --- | --- | --- | --- | --- |
| 1 | J. N. Fan, 2020[8] | 50, M | spinal neurofibroma,  parotid gland mass,  recurrent shingles | on admission | 211/NA | never | CSF VZV PCR,  brain biopsy | none | NA | yes | none | cured |
| 2 | A. Lau,  2020[9] | 34, M | retinopathy, MAC, IRIS | none | a last known 45/NA | 1 month | CSF VZV PCR | HIV cardiomyopathy. | NA | yes | yes | improving |
| 3 | M. F. Granja, 2019[10] | 56, M | HTN, stroke | none | 25/501,000 | never | CSF VZV PCR | PCP, MAC | 3 weeks | yes | none | improving |
| 4 | J. A. Deviley, 2019[11] | 5,  F | chickenpox, VZV | 9 month before | 6/NA | 7 years | CSF VZV PCR, tissue VZV PCR | PS deficiency | about  2 years | yes | none | dead |
| 5 | Ye C., 2019 [12] | 49, M | HTN, VZV encephalopathy | NA | 66/NA | NA | CSF VZV IgG/IgM | none | more than 2 weeks | yes | yes | improving |
| 6 | Handoko R., 2019[13] | 48, M | pneumothorax, MRSA bacteremia,  gastrointestinal bleeding | NA | 2/66 | 2 weeks | CSF VZV PCR | cerebral hemorrhage | NA | yes | none | dead |
| 7 | Kristin R V Harrington, 2019[14] | 35, M | HIV encephalopathy | none | 155/NA | 10 weeks | CSF VZV IgG | cerebral hemorrhage, left retinal detachment | NA | yes | none | improving |
| 8 | A. Tomkins, 2018[15] | 38,  F | none | none | 24/1,350,259 | never | CSF/serum VZV IgG | none | NA | yes | yes | cured |
| 9 | Messe M., 2018[16] | 36, F | none | none | 144/>1.000.000 | never | CSF VZV PCR | none | NA | yes | none | improving |
| 10 | Lopes M., 2018[17] | 37, F | none | none | NA/ >1 M copies/lL | never | CSF VZV PCR | none | 3 weeks | yes | yes | improving |
| 11 | Villanueva F., 2018[18] | 24, M | cutaneous Kaposi's sarcoma | none | 1/NA | never | CSF VZV PCR | none | 3 weeks | none | none | dead |
| 12 | R. A. B. Ignacio, 2017 [19] | 31, F | chickenpox, VZV, CMV CNS infection | within a month | 245/undetectable | about 6 month | CSF VZV PCR | none | 6 weeks | yes | yes | improving |
| 13 | Alkhalifah M., 2016[20] | 25, M | none | none | 33/NA | never | CSF VZV PCR | none | 2 weeks | yes | none | improving |
| 14 | A. Lefkowitz, 2015[21] | 56, F | PCP, chickenpox, HBV, idiopathic retinal vasculitis | on admission | 72/NA | never | CSF VZV PCR | none | NA | NA | NA | NA |
| 15 | Vela-Duarte D. 2015[22] | 48, M | HCV | none | 72/577,005 | never | CSF VZV PCR | cerebral hemorrhage | NA | yes | none | improving |
| 16 | S. Y. Teo, 2014[23] | 36, F | TB, VZV meningitis? | 3 week before | 179/2,300 | 1 month | CSF VZV PCR | VZV IRIS  (VZV CSF PCR negative) | NA | yes | yes (for IRIS) | cured |
| 17 | Agnihotri S., 2014  [24] | 30, F | none | none | NA/NA | NA | CSF VZV PCR | NA | NA | NA | NA | NA |
| 18 | Kraus M., 2014[25] | 35, M | syphilis | none | NA/NA | 2 month before | CSF VZV PCR | neurosyphilis | NA | yes | yes | NA |
| 19 | Philip J., 2013 | 27, M | NA | NA | 54/NA | NA | CSF VZV IgG | NA | NA | yes | none | dead |
| 20 | M. A. Iro, 2013[26] | 11, F | chickenpox, pneumoniae | none | 30/404 | 1 month | CSF VZV PCR | none | 7 months | yes | none | improving |
| 21 | C. Yasuda, 2013[27] | 35, M | syphilis | none | 116/undetected on day 15 | 5 months | CSF VZV IgG index | none | 6 weeks | yes | none | improving |
| 22 | S. C. Sasson, 2013[28] | 54, M | anal squamous  cell carcinoma, liver metastasis | on admission | 173/36 | never | CSF VZV PCR | PRES,  CSF-EBV positive | 2 weeks | yes | yes | improving |
| 23 | Satyan S., 2012[29] | 62, M | none | within a week | 700/NA | NA | CSF VZV PCR | none | NA | NA | NA | NA |
| 24 | A. Stanley, 2012[30] | 22, F | CMV, VZV retinitis | none | 29/2,037, 280 | about one year | CSF VZV PCR,  IgG index | retinitis | 2 weeks | yes | none | blind as a consequence  of CMV retinitis |
| 25 | D. Vibha, 2012[31] | 48, F | none | on admission | 211/NA | never | CSF VZV IgG | none | 2 weeks | yes | yes | cured |
| 26 | J. Gutierrez, 2011[32] | 36, M | PFO | NA | 22/438 | never | CSF VZV IgG | NA | none | NA | NA | rehabilitation |
| 27 | J. Gutierrez, 2011[32] | 46, M | hyperhomocysteinemia anticardiolipin antibody (+) PC/PS hypoactivity lupus anticoagulant(+) | NA | 76/NA | never | CSF VZV PCR | NA | none | NA | NA | nursing home |
| 28 | J. Gutierrez, 2011[32] | 24,  F | dyslipidemia | NA | 15/0 | never | CSF VZV PCR | NA | none | NA | NA | nursing home |
| 29 | J. Gutierrez, 2011[32] | 37, F | dyslipidemia | NA | 149/555,340 | recently initiated | CSF VZV PCR | NA | NA | NA | NA | rehabilitation |
| 30 | J. Gutierrez, 2011[32] | 17, F | none | NA | 3/392,978 | stopped months prior to the stroke | lung VZV PCR | NA | NA | NA | NA | dead |
| 31 | J. Mareedu, 2011[33] | 35, M | histoplasmosis, asthma, HTN | none | 44/31, 527 | 9 years | CSF VZV PCR | none | 8 months | yes | none | cured |
| 32 | S. D. Newsome, 2009[34] | 42, F | none | on admission | 3/53,085 | 2 weeks | CSF VZV PCR | none | for 2 weeks, but more than 2 month at relapse | yes | started on  second episode | relapsed,  but improving in second episode |
| 33 | H. A. Metta, 2009[35] | 32, M | left HZO | 4 month before | 59/>500 000 | two weeks | clinically  (PCR, antibody negative) | none | 3 weeks | yes | yes | improving |
| 34 | C. C. Chang, 2009[36] | 41, F | none | on admission | 83/NA | changed ART due to virological failure two weeks before | CSF VZV PCR | myelitis, meningoencephalitis | 60 hour | yes | yes | dead |
| 35 | G. A. Ortiz, 2008[37] | 24, F | recently HZO | 2 week before | 10/NA | never | CSF VZV PCR | encephalomalacia  within the pons | more than  8 weeks | yes | yes | cured |
| 36 | M. A. Nagel, 2007[38] | 52, M | NA | on admission | NA/NA | NA | CSF VZV PCR, IgG | NA | NA | yes | none | improving |
| 37 | T. Saraya, 2006[39] | 36, M | none | on admission | 109/39,000 | NA | CSF VZV PCR | none | 4 weeks | yes | yes | cured |
| 38 | H. Takeoka, 2006[40] | 43, M | chickenpox, Ramsay-hunt syndrome | 1 month before | 169/96,000 | never | plasma/auricular  VZV PCR |  | 2 months | yes | none | cured |
| 39 | A. K. Patel, 2006[41] | 35, M | TB | 20 day before | 228/NA | 10 months | CSF VZV IgG, IgM | none | NA | yes | none | cured |
| 40 | G. L. de la Grandmaison, 2005[42] | 46, M | VZV | more than 1 year  before | 4/102,000 | 7 years | CSF VZV PCR | none | NA | yes | none | dead |
| 41 | C. Amlie-Lefond, 1995[43] | 30, M | VZV | 4 month before | 4/NA | none | CSF VZV PCR | cerebral hemorrhage | NA | yes | yes | improving |
| 42 | C. Amlie-Lefond, 1995[43] | 29, M | VZV | 8 month before | NA/NA | none | autopsy | none | NA | yes | none | dead |
| 43 | C. Amlie-Lefond, 1995[43] | 33, M | VZV | NA | NA/NA | none | autopsy | encephalitis, hydrocephalus | NA | NA | NA | dead |
| 44 | C. Amlie-Lefond,1995[43] | 37, M | VZV | NA | NA/NA | none | autopsy | encephalitis | NA | NA | NA | dead |
| 45 | I. Corral, 2003[44] | 35, M | VZV | 1 year before | 390/NA | NA | CSF VZV PCR | none | NA | yes | none | severe  neurological deficit |
| 46 | A. Kronenberg, 2002[45] | 28, M | HCV | none | 13/92,029 | two weeks | CSF VZV PCR, IgG | none | 7 weeks | yes | none | cured |
| 47 | J. Berkefeld, 2000[46] | 31, M | recurrent TIA, right hemiparesis | none | NA/NA | NA | serum VZV PCR, IgG | NA | 3 weeks | yes | yes | cured |
| 48 | D. H. Gilden, 1998[47] | 34, M | VZV | 6 month before | NA/NA | NA | CSF VZV PCR, IgG | none | NA | yes | none | improving |
| 49 | D. H. Gilden, 1998[47] | 54, M | malignant melanoma,  HBV, oral herpes | none | 305/NA | NA | CSF VZV PCR,  CSF VZV IgG/IgM | hydrocephalus, encephalitis | 2 weeks | yes | yes | dead |
| 50 | B. B. Fulmer, 1998[48] | 6,  F | none | none | NA/NA | none | autopsy | SAH, hydrocephalus,  multiple aneurysm | none | none | none | dead |
| 51 | Aygun N, 1998[49] | 41, M | PCP, systemic parvovirus infection | none | 20/NA | NA | brain biopsy | bilateral retinitis | NA | yes | none | improving |
| 52 | B. K. Kleinschmidt- DeMasters, 1998[50] | 54,  M | alcohol abuse, malignant melanoma, HBV, oral herpes | none | 304/NA | NA | CSF VZV PCR,  IgG/IgM | hydrocephalus, multiple foci of acute subpial hemorrhage | NA | yes | none | dead |
| 53 | Y. Frank, 1997[51] | 8,  F | none | on admission | 11/NA | NA | CSF VZV PCR | herniation syndrome | 10 weeks | yes | none | dead |
| 54 | O. Picard, 1997[52] | 31,  NA | none | none | 2/NA | NA | CSF VZV PCR | HZO | 1 month | yes | yes | cured |
| 55 | O. Picard, 1997[52] | 45,  NA | none | none | 155/NA | NA | CSF VZV PCR | HZO | 1 month | yes | yes | improving |
| 56 | L. C. Kenyon, 1996[53] | 57,  M | PCP | none | 40/NA | none | autopsy | radiculomyelitis | NA | yes | yes | dead |
| 57 | BK. Kleinschmidt-DeMasters, 1996[50] | 29,  M | VZV | 8 month before | NA/NA | none | autopsy | none | 2 days | yes | none | dead |
| 58 | BK. Kleinschmidt-DeMasters, 1996[50] | 36,  M | VZV | 2 month before | NA/NA | none | autopsy | none | none | none | none | dead |
| 59 | BK. Kleinschmidt-DeMasters, 1996[50] | 33,  M | genital herpes, VZV, sinusitis,  esophageal candidiasis, Giardia infection, streptococcal pneumonitis, cryptosporidiosis, HBV | on admission | NA/NA | none | autopsy | hydrocephalus | none | none | none | dead |
| 60 | BK. Kleinschmidt-DeMasters, 1996[50] | 17,  M | hemophilia A, chiekenpox, VZV | 6 month before | NA/NA | 6 years | autopsy | none | none | none | none | dead |
| 61 | NEJM, 1996[54] | 37,  M | PCP, disseminated VZV | 10 month before | 16/NA at 9 month before | 38 month | autopsy | cerebral hemorrhage | 1 month | yes | none | dead |
| 62 | G. J. van den Horn, 1996[55] | 36,  M | right HZO | 3 month before | 20/NA | none | autopsy | PORN | more than  2 years | yes | none | dead |
| 63 | L. C. Kenyon, 1996[53] | 57,  M | PCP | NA | 40/NA | NA | autopsy | ventriculo-encephalitis, vasculitis with spinal infarction | within a month | foscarnet | none | dead |
| 64 | H Manji, 1995[56] | 55,  M | no | 3 week before | NA/NA | none | clinically | left HZO | NA | yes | none | NA |
| 65 | F. Gray, 1994[57] | 34,  F | drug addict | on admission | NA/NA | NA | autopsy | none | NA | NA | NA | dead |
| 66 | F. Gray, 1994[57] | 39,  F | drug addict | on admission | 12/NA | NA | autopsy | none | NA | NA | NA | dead |
| 67 | F. Gray, 1994[57] | 29,  M | none | on admission | 44/NA | NA | autopsy | none | NA | NA | NA | dead |
| 68 | F. Gray, 1994[57] | 57,  M | none | none | NA/NA | NA | autopsy | multinucleated  giant cell? | NA | NA | NA | dead |
| 69 | F. Rousseau, 1993[58] | 33,  M | chickenpox | within a year | 20/NA | none | NA | bilateral retinal necrosis | NA | NA | NA | dead |
| 70 | F. Rousseau, 1993[58] | 54,  F | chickenpox | within a year | 23/NA | none | NA | right retinal necrosis | NA | yes | none | dead |
| 71 | F. Rousseau, 1993[58] | 28,  M | chickenpox | within a year | 26/NA | none | brain biopsy | occlusion of the right central retinal artery | NA | yes | none | lost follow up |
| 72 | F Chrétien, 1993  [59] | 30,  M | none | none | 290/NA | none | autopsy | HIV encephalitis, ventriculitis | none | none | none | dead |
| 73 | F. Gray, 1992  [60] | 34,  F | IVD | 7 month before | NA/NA | NA | autopsy | encephalitis | none | yes | none | dead |
| 74 | Y. Frank, 1989  [61] | 4.5,  F | chronic hepatitis, lymphoid interstitial pneumonia, chickenpox dysplasia-involution of the thymus | 1 year before | 520/NA | none | autopsy | NA | NA | yes | yes | dead |
| 75 | H V Vinters, 1988[62] | 40,  M | syphilis, gonorrhea | none | <200/NA | none | autopsy | myelitis | NA | NA | yes | dead |
| 76 | S. Morgello, 1988[63] | 29,  M | VZV, E. histolytica, ITP, PCP, retinitis | 2 month before | NA/NA | none | autopsy | PCP, MAC | NA | NA | NA | dead |
| 77 | our case, 2020 | 55,  M | diabetes, shingles | 2 month before | 48.8/240, 000 | two week | CSF VZV PCR | cerebral hemorrhage, IRIS | continue | yes | yes | severe neurological deficit |

Abbreviation: M, male; F, female; VZV, Varicella-zoster virus; ART, antiretroviral therapy; ACV, acyclovir; FOS, foscarnet; GCV, ganciclovir; GACV, valacyclovir; NA, not available; CSF, cerebral spinal fluid; PCR, polymerase chain reaction; MAC, mycobacterium avium complex; IRIS, immune reconstitution inflammatory syndrome; HIV, human immunodeficiency virus; PCP, pneumocystis pneumoniae; HTN, hypertension; MRSA, methicillin-resistant *Staphylococcus aureus*; CNS, central nerve system; CMV, Cytomegalo virus; HBV, hepatitis B virus; HCV, hepatitis C virus; TB, *mycobacterium tuberculosis*; PFO, patent foramen ovale; HZO, herpes zoster ophthalmicus; SAH, Sub-arachnoid hemorrhage; TIA, transient ischemic attack

Figure S1: The process of the literature review regarding crushed BIC/TAF/FTC


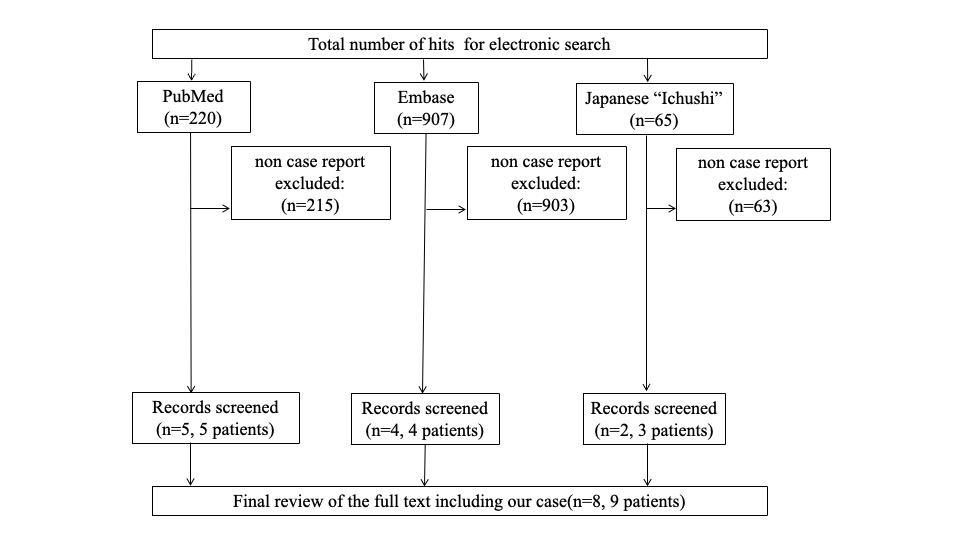


Abbreviations: BIC/TAF/FTC, bictegravir/emtricitabine/tenofovir alafenamide

Figure S2: The process of the literature review for Varicella-zoster virus vasculopathy with stroke in patients with HIV


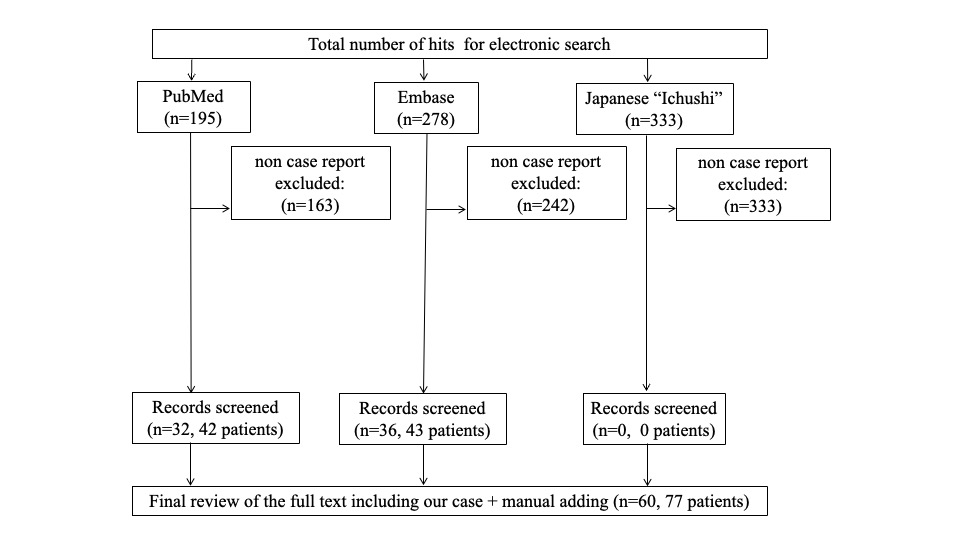


Abbreviations: HIV, human immunodeficiency virus

**Reference**

1. Fulco, P.P., *Crushed bictegravir/emtricitabine/tenofovir alafenamide in a human immunodeficiency virus-positive patient with esophageal cancer.* Am J Health Syst Pharm, 2020. **77**(7): p. 509-510.

2. Lozano, A.B., et al., *Failure to bictegravir and development of resistance mutations in an antiretroviral-experienced patient.* Antiviral Res, 2020. **179**: p. 104717.

3. Ferrandez, J.S., et al., *Successful Bictegravir/Emtricitabine/Tenofovir Alafenamide Treatment in a HIV Patient With Swallowing Difficulties.* Ann Pharmacother, 2021. **55**(4): p. 556-557.

4. Rowe, S.M., et al., *Increased viral load in a hospitalized patient on treatment with crushed bictegravir/emtricitabine/tenofovir alafenamide: A case report and review of the literature.* Am J Health Syst Pharm, 2022. **79**(16): p. 1330-1336.

5. Roa, P.E. and R. Bazzi, *Crushed bictegravir/emtricitabine/tenofovir alafenamide in a human immunodeficiency virus-positive patient with pancreatic cancer.* Int J STD AIDS, 2022. **33**(1): p. 97-98.

6. Rie M., et al., *Two cases in which bictegravir was administered by the simple suspension method and plasma bictegravir concentrations were studied*[Japanese]*.* *The Japanese Society for AIDS Research*, 2020. **22**(4): p. 491.

7. Kazuyoshi M., *A case of therapeutic effect achieved by crushing BIC/FTC/TAF combination tablet*s[Japanese]*. The Japanese Society for AIDS Research*,, 2020. **22**(4): p. 488.

8. Fan, J.N., et al., *Varicella-zoster virus causing a ring-like cerebral lesion in AIDS.* Proc (Bayl Univ Med Cent), 2020. **33**(4): p. 614-615.

9. Lau, A., E.O. Essien, and I.J. Tan, *Zoster Sine Herpete Masquerading as Central Nervous System Vasculitis.* Cureus, 2020. **12**(3): p. e7231.

10. Granja, M.F., et al., *HIV vasculopathy versus VZV vasculitis in an HIV patient with multiple brain ischaemic infarcts.* BMJ Case Rep, 2019. **12**(7).

11. Deviley, J.A., et al., *Recurrent strokes, central nervous system vasculitis, and acquired protein S deficiency secondary to varicella zoster in a child with AIDS.* J Neurovirol, 2019. **25**(1): p. 137-140.

12. Ye, C. and A. Anderson, *For stroke's sake: Varicella zoster virus (VZV) vasculopathy.* Journal of General Internal Medicine, 2019. **34**(2): p. S535.

13. Handoko, R., et al., *Varicella zoster virus (VZV) vasculopathy as a potential cause of altered mental status in an immu-nocompromised host.* Journal of General Internal Medicine, 2019. **34**(2): p. S679-S680.

14. Harrington, K.R.V., P. Rhyner, and R.R. Kempker, *Hospital Onset Varicella Central Nervous System Vasculitis in a Patient with HIV Infection.* AIDS Res Hum Retroviruses, 2019. **35**(4): p. 357-358.

15. Tomkins, A., et al., *Varicella zoster virus cerebral aneurysmal vasculopathy presenting in a newly-diagnosed HIV-positive patient.* Int J STD AIDS, 2018. **29**(13): p. 1351-1353.

16. Messe, M. and E. Carrera, *Postvaricella vasculopathy presenting with acute ischemic stroke and proximal intracranial vessel dissection.* European Journal, 2018. **3**(1): p. 425.

17. Lopes, M., et al., *Arterial invasion - Complication of a common virus.* European Stroke Journal, 2018. **3**(1): p. 585-586.

18. Villanueva, F., et al., *Varicella zoster virus vasculopathy: A case of cere-brovascular accident in an immunosuppressed patient.* Journal of General Internal Medicine, 2018. **33**(2): p. 659.

19. Bender Ignacio, R.A., et al., *T Cell Immunity to Varicella-Zoster Virus in the Setting of Advanced HIV and Multiple Varicella-Zoster Virus Recurrences.* Viral Immunol, 2017. **30**(1): p. 77-80.

20. Alkhalifah, M., et al., *Varicella zoster vasculopathy presenting with multifocal fusiform aneurysms.* Interventional Neurology, 2016. **5**: p. 15.

21. Lefkowitz, A., et al., *Multifocal strokes in a 56-year-old man with HIV infection.* CMAJ, 2015. **187**(14): p. 1067-1070.

22. Vela-Duarte, D., D. Pasquale, and M. Flaster, *Varicella-zoster hemorrhagic encephalitis in an Aids patient.* Neurology, 2015. **84**.

23. Teo, S.Y., et al., *Central nervous system-immune reconstitution inflammatory syndrome presenting as varicella zoster virus-mediated vasculitis causing stroke.* Int J STD AIDS, 2014. **25**(9): p. 683-5.

24. Agnihotri, S. and N. Venna, *Atypical neurologic complications of varicella zoster virus.* Journal NeuroVirology, 2013. **19**: p. S1-S2.

25. Kraus, M., *Varicella zoster virus vasculitis in an HIV-positive male presenting with stroke.* Journal of General Internal Medicine, 2012. **27**: p. S499.

26. Iro, M.A., et al., *Varicella zoster virus central nervous system immune reconstitution inflammatory syndrome presenting in a child.* Pediatr Infect Dis J, 2013. **32**(11): p. 1283-4.

27. Yasuda, C., et al., *[Cerebral infarction and intracranial aneurysm related to the reactivation of varicella zoster virus in a Japanese acquired immunodeficiency syndrome (AIDS) patient].* Rinsho Shinkeigaku, 2013. **53**(9): p. 701-5.

28. Sasson, S.C., et al., *Posterior reversible encephalopathy syndrome (PRES) in an HIV-1 infected patient with disseminated varicella zoster virus: a case report.* BMC Infect Dis, 2013. **13**: p. 396.

29. Satyan, S., A. Bragdon, and B. Jubelt, *Ramsey hunt syndrome with dysphagia with MRI evidence of brain stem and upper cervical cord involvement.* Neurology, 2012. **78**(1).

30. Stanley, A., et al., *The complexity of HIV vasculopathy.* S Afr Med J, 2012. **102**(6): p. 474-6.

31. Vibha, D., et al., *Varicella zoster vasculopathy presenting as lateral medullary syndrome.* J Neurovirol, 2012. **18**(6): p. 538-40.

32. Gutierrez, J. and G. Ortiz, *HIV/AIDS patients with HIV vasculopathy and VZV vasculitis: a case series.* Clin Neuroradiol, 2011. **21**(3): p. 145-51.

33. Mareedu, J., et al., *Varicella zoster vasculopathy.* J Int Assoc Physicians AIDS Care (Chic), 2011. **10**(3): p. 144-5.

34. Newsome, S.D. and A. Nath, *Varicella-zoster virus vasculopathy and central nervous system immune reconstitution inflammatory syndrome with human immunodeficiency virus infection treated with steroids.* J Neurovirol, 2009. **15**(3): p. 288-91.

35. Metta, H.A., et al., *Difficulties in the diagnosis of delayed contralateral hemiparesis due to varicella-zoster virus in an HIV-positive patient.* Int J Infect Dis, 2009. **13**(5): p. e329-30.

36. Chang, C.C., et al., *Fatal acute varicella-zoster virus hemorrhagic meningomyelitis with necrotizing vasculitis in an HIV-infected patient.* Clin Infect Dis, 2009. **48**(3): p. 372-3.

37. Ortiz, G.A., et al., *Ramsay hunt syndrome followed by multifocal vasculopathy and posterior circulation strokes.* Neurology, 2008. **70**(13): p. 1049-51.

38. Nagel, M.A., et al., *The value of detecting anti-VZV IgG antibody in CSF to diagnose VZV vasculopathy.* Neurology, 2007. **68**(13): p. 1069-73.

39. Saraya, T., et al., *Evidence for vascular spread of varicella zoster-associated vasculopathy.* Ann Intern Med, 2006. **144**(7): p. 535-7.

40. Takeoka, H., et al., *[Varicella-zoster virus symptoms and polyneuropathy in a patient with human immunodeficiency virus infection not improved until highly active anti-retroviral therapy added to acyclovir therapy].* Kansenshogaku Zasshi, 2006. **80**(1): p. 46-50.

41. Patel, A.K., et al., *Immune reconstitution syndrome presenting with cerebral varicella zoster vasculitis in HIV-1-infected patient: a case report.* J Int Assoc Physicians AIDS Care (Chic), 2006. **5**(4): p. 157-60.

42. de la Grandmaison, G.L., et al., *"Burnt out" varicella-zoster-virus encephalitis in an AIDS patient following treatment by highly active antiretroviral therapy.* Clin Radiol, 2005. **60**(5): p. 613-7.

43. Amlie-Lefond, C., et al., *The vasculopathy of varicella-zoster virus encephalitis.* Ann Neurol, 1995. **37**(6): p. 784-90.

44. Corral, I., et al., *Neurological complications of varicella-zoster virus in human immunodeficiency virus-infected patients: changes in prevalence and diagnostic utility of polymerase chain reaction in cerebrospinal fluid.* J Neurovirol, 2003. **9**(1): p. 129-35.

45. Kronenberg, A., et al., *Multifocal vasculopathy due to Varicella-Zoster Virus (VZV): serial analysis of VZV DNA and intrathecal synthesis of VZV antibody in cerebrospinal fluid.* Clin Infect Dis, 2002. **35**(3): p. 330-3.

46. Berkefeld, J., W. Enzensberger, and H. Lanfermann, *MRI in human immunodeficiency virus-associated cerebral vasculitis.* Neuroradiology, 2000. **42**(7): p. 526-8.

47. Gilden, D.H., et al., *The value of cerebrospinal fluid antiviral antibody in the diagnosis of neurologic disease produced by varicella zoster virus.* J Neurol Sci, 1998. **159**(2): p. 140-4.

48. Fulmer, B.B., et al., *Two cases of cerebral aneurysms in HIV+ children.* Pediatr Neurosurg, 1998. **28**(1): p. 31-4.

49. Aygun, N., et al., *Multifocal varicella-zoster virus leukoencephalitis in a patient with AIDS: MR findings.* AJNR Am J Neuroradiol, 1998. **19**(10): p. 1897-9.

50. Kleinschmidt-DeMasters, B.K., et al., *Profound cerebrospinal fluid pleocytosis and Froin's Syndrome secondary to widespread necrotizing vasculitis in an HIV-positive patient with varicella zoster virus encephalomyelitis.* J Neurol Sci, 1998. **159**(2): p. 213-8.

51. Frank, Y., et al., *Childhood AIDS, varicella zoster, and cerebral vasculopathy.* J Child Neurol, 1997. **12**(7): p. 464-6.

52. Picard, O., et al., *Cerebral infarction associated with vasculitis due to varicella zoster virus in patients infected with the human immunodeficiency virus.* Biomed Pharmacother, 1997. **51**(10): p. 449-54.

53. Kenyon, L.C., et al., *Varicella-zoster ventriculo-encephalitis and spinal cord infarction in a patient with AIDS.* Acta Neuropathol, 1996. **92**(2): p. 202-5.

54. *Case records of the Massachusetts General Hospital. Weekly clinicopathological exercises. Case 36-1996. A 37-year-old man with AIDS, neurologic deterioration, and multiple hemorrhagic cerebral lesions.* N Engl J Med, 1996. **335**(21): p. 1587-95.

55. van den Horn, G.J., C. Meenken, and D. Troost, *Association of progressive outer retinal necrosis and varicella zoster encephalitis in a patient with AIDS.* Br J Ophthalmol, 1996. **80**(11): p. 982-5.

56. Manji, H., et al., *Movement disorders in AIDS: Infective, neoplastic and iatrogenic causes.* Parkinsonism Relat Disord, 1995. **1**(1): p. 13-9.

57. Gray, F., et al., *Varicella-zoster virus infection of the central nervous system in the acquired immune deficiency syndrome.* Brain, 1994. **117 ( Pt 5)**: p. 987-99.

58. Rousseau, F., et al., *Necrotizing retinitis and cerebral vasculitis due to varicella-zoster virus in patients infected with the human immunodeficiency virus.* Clin Infect Dis, 1993. **17**(5): p. 943-4.

59. Chretien, F., et al., *Acute varicella-zoster virus ventriculitis and meningo-myelo-radiculitis in acquired immunodeficiency syndrome.* Acta Neuropathol, 1993. **86**(6): p. 659-65.

60. Gray, F., et al., *Varicella-zoster virus encephalitis in acquired immunodeficiency syndrome: report of four cases.* Neuropathol Appl Neurobiol, 1992. **18**(5): p. 502-14.

61. Frank, Y., et al., *Multiple ischemic infarcts in a child with AIDS, varicella zoster infection, and cerebral vasculitis.* Pediatr Neurol, 1989. **5**(1): p. 64-7.

62. Vinters, H.V., et al., *Necrotizing vasculitis of the nervous system in a patient with AIDS-related complex.* Neuropathol Appl Neurobiol, 1988. **14**(5): p. 417-24.

63. Morgello, S., et al., *Varicella-zoster virus leukoencephalitis and cerebral vasculopathy.* Arch Pathol Lab Med, 1988. **112**(2): p. 173-7.
